# Co‐Designing an Engagement Strategy to Include the Voices of a Minority Group in Assessing the Quality of Maternity and Neonatal Care

**DOI:** 10.1111/hex.70376

**Published:** 2025-08-11

**Authors:** Thérèse McDonnell, Jaspreet Kaur Dullat, Louise Hendrick, Léan McMahon, Gemma Moore, Emily Murphy, Carmen Nae, Danut Nae, Marianna Prontera, Eilish McAuliffe

**Affiliations:** ^1^ Centre for Interdisciplinary Research, Education and Innovation in Health Systems (IRIS), UCD School of Nursing, Midwifery and Health Systems Dublin Ireland; ^2^ National Quality & Patient Safety, Health Service Executive (HSE) Dublin Ireland; ^3^ National Women and Infants Health Programme (NWIHP), Health Service Executive (HSE) Dublin Ireland; ^4^ Roma Programme, Cairde Dublin Ireland

**Keywords:** co‐design, experience, maternity services, minority, quality, Roma

## Abstract

**Introduction:**

This study outlines the co‐design approach taken to engagement with women from the Roma community to capture their experience of maternity and neonatal care in Ireland. The findings of this study will be used to assess the relevance and completeness of measures of quality and safety of maternity care in capturing these experiences.

**Methods:**

An engagement strategy was co‐designed with Cairde, a community health development organisation. A series of four workshops were co‐designed to facilitate open discussion with women from the Roma community (*n* = 8) on their experiences and perspectives of maternity and neonatal care in Ireland and to provide information to participants on the progression of pregnancy, preparation for childbirth, their health and healthcare access. The co‐design approach aimed to identify and address barriers to effective engagement, including mistrust, experiences of discrimination, accessibility, literacy and language proficiency and to ensure the workshops were delivered in a culturally sensitive manner. A questionnaire to capture details of their experience of accessing and using maternity services was also co‐designed with Cairde, whose Roma Programme staff facilitated data collection.

**Results:**

The experience of women from the Roma community using Irish maternity services from pregnancy to childbirth and postnatal care was captured. The involvement of Cairde gained the trust of participants, facilitated open discussion and ensured full engagement throughout. The use of pictures/graphics and verbal communication with non‐technical language, with translation and interpretation by Roma Peer Support Workers, ensured the online workshops were accessible. Online delivery was made possible within the existing digital framework of Cairde's Roma Programme.

**Conclusions:**

Through partnering with Cairde, a community health development organisation with the capacity to participate and strong links to the Roma community, the many barriers to engagement with this marginalised minority community were addressed. This partnership approach afforded the research team privileged access to the Roma community, facilitating the team to engage with this seldom‐heard group.

**Patient or Public Contribution:**

This collaboration between academic researchers, staff from the Health Service Executive (HSE), and Cairde, a community health development organisation, aimed to involve all stakeholders, including members of the Roma community, in the co‐design of an engagement strategy that allowed the experiences and perspectives of women from the Roma community who have given birth in Ireland to be captured. Staff from Cairde, including members of the Roma Community, co‐designed a series of workshops and a questionnaire, and provided support on the set‐up and delivery of the workshops and questionnaire. Two of the eight Roma women who participated in the co‐design process also participated in the workshops. They provided guidance on communication and cultural considerations. This collaboration enabled the successful delivery of focus groups, informational sessions and a questionnaire, with full participation by the eight participants. Staff from Cairde, including members of the Roma community, also contributed to the interpretation of findings and drafting of the papers. Partnering with Cairde afforded privileged access to the Roma community, allowing the research team to engage with this seldom‐heard group. The many barriers to this engagement were addressed through partnering with an organisation with the trust of the Roma community and with the capacity to participate [1].

## 1 Background

Measuring quality in healthcare is challenging due to its multifaceted and complex construction [2, 3, 4]. Measures should reflect what is important to all stakeholders, including patient experience, and integrate this experience with outcome and process measures into a balanced suite of quality indicators [3]. This approach can improve accountability, which is particularly important in the measurement of the quality of maternity services, where poor quality care provision can have catastrophic consequences [5, 6]. The quality of care for women and newborns is defined by the World Health Organisation (WHO) as ‘the degree to which health services (for individuals and populations) increase the likelihood of timely, appropriate care for the purpose of achieving desired outcomes that are both consistent with current professional knowledge and take into account the preferences and aspirations of individual women and their families’ [7]. The WHO further expands on this definition to specify the inclusion of two important components of care, ‘the quality of the provision of care and the quality of care as experienced by women, newborns and their families’. Members of the public are users of health services and can provide valuable information about what works in practice and what does not.

In capturing the experiences and perceptions of service users, engagement should reflect the diversity of women who use maternity services. Engagement with communities with poorer health outcomes and for whom there are known inequities in healthcare provision is critical to building an understanding of the drivers of such inequity and to designing mechanisms to address this variation in health outcomes. Pregnant women who are recent migrants to well‐resourced countries, those with lower socioeconomic status, and temporary housing are at increased risk for poor health outcomes and reduced access to high‐quality care [8, 9]. The absence of care that is culturally appropriate and accommodates the needs, preferences and aspirations of individuals can also affect their decisions to engage with skilled maternity care [10, 11, 12].

The Roma community experiences significant disadvantage across all measures of socioeconomic status. As the largest and most marginalised ethnic minority group in Europe, this community experiences discrimination in many areas of life [13, 14]. The 2022 Census reported that 16,059 Roma live in Ireland; however, it is widely acknowledged that Roma throughout Europe, including Ireland, are dramatically undercounted [15]. A recent report drawing on the Equality Attitudes Survey 2023 identified significant negative sentiment and racism towards Roma in Ireland [16]. The EU Commission describes a lack of trust between Roma and mainstream society in general [14, 17]. Roma people in Ireland mistrust health services and have difficulty accessing healthcare due to discrimination, cost, cultural barriers and language [14, 17, 18], and therefore can be categorised as ‘seldom‐heard’ – a term used to describe groups who may experience barriers to accessing services or are underrepresented in healthcare decision making. Their identity through dress, social and cultural beliefs, often distinguishes Roma women from the majority of the Irish population and contributes to marginalisation [14, 18, 19]. Roma women tend to marry young, often younger than 20 years, and have a high birth rate [20]. According to the 2014 Roma health report, the 2011 survey identified that the average age of childbirth for Roma in Europe was 17.27 years, with the highest birth rate among the most socially disadvantaged Roma families [17]. It has been reported that 24% of pregnant Roma women first attend maternal health services when giving birth [19]. The low utilisation of antenatal services and the high number of births to teenage mothers puts Roma women and their children at increased risk of complications [14]. Given their marginalised status and high birth rate, coupled with an increased risk of obstetric complications, incorporating the perceptions and experiences of Roma women in the design of a national suite of measures of the quality of maternity services will enhance the relevance of measures and ensure they represent the diversity of those using the Irish maternity services.

Ocloo and colleagues stress the importance of including diverse populations in the co–design research process; however, they also acknowledge the many barriers to achieving genuine public and patient involvement [21, 22]. Participants may lack proficiency in the native language [23] and may therefore be less confident or unwilling to engage. Literacy issues can mean that the correspondence and paperwork involved in preparing and attending meetings may be problematic to some participants. Health literacy [24], in particular the use of technical terms and jargon, may result in participants not volunteering or not remaining engaged with research or service improvement activities. The lack and cost of transportation may also preclude attendance [25]. Caring responsibilities, timing of meetings and limited free time all present additional challenges [25]. Furthermore, mistrust can be a significant impediment to engaging minority communities in research, particularly clinical research.

When minority groups are excluded from public and patient involvement (PPI) activities, they are denied the opportunity to shape improvements to healthcare that would benefit their lives [26, 27]. Targeted approaches are required to involve specific groups, including marginalised groups, to overcome barriers of low literacy, communication and language difficulties [22]. However, gaining the trust of minority communities can be one of the biggest hurdles to their engagement. While there is uncertainty about how to do PPI engagement effectively, i.e., in ways that constitute genuine partnerships and which involve a diversity of participants [21], a rapid realist review conducted by Ní Shé et al. (2019) identified a number of critical mechanisms for successful engagement with seldom‐heard groups. A key conclusion from this review was the importance of reciprocity by engaging participants from the start of the research project and using methods such as co‐design, co‐production and emancipatory research. Ní Shé et al. argue ‘as the shift away from a “fund and forget model” continues, the need to resource pre‐engagement and long‐term partnerships grows’ and they call for further contributions to the literature on reciprocal projects with seldom‐heard groups [28]. Our study responds to this call by partnering with a community health development organisation that is actively working with and supporting minority communities: Cairde. This partnership approach aimed to co‐design an engagement strategy that captured the contribution of a seldom‐heard group. Community organisations often act as a bridge between state bodies and the community, have the trust of those they support, and the tools and understanding to facilitate engagement in research and service improvement activities they believe are in the interests of the community they serve.

The engagement process described in this paper forms part of a larger study which aims to develop a suite of measures of the quality and safety of maternity and neonatal services in Ireland [29]. The measure identification process involved a review of literature and measures adopted by health services internationally, a co‐design process with a Clinical Advisory Group, and engagement with a panel of service user representatives. Participation in the representative panel required a high level of literacy and confidence with clinical terminology; consequently, this process was not accessible to many potential participants [30]. An engagement strategy was therefore designed to involve women from a marginalised community who may‐ have been unable to contribute to this panel. As many minority communities are culturally distinct and do not share the same language, it was not appropriate to invite women from different minority communities to participate in one combined focus group discussion (FGD). Due to their marginalised status, low level of engagement with maternity services during the antenatal period, and increased risk of obstetric complications, the Roma community was identified as an important contributor to this process.

The aim of this study was to design an engagement strategy that allowed the experiences and perspectives of women from the Roma community who have given birth in Ireland to be captured in a manner that allowed for assessment of the relevance and completeness of a suite of measures of quality of maternity care.

## 2 Materials and Methods

**Figure 1:**
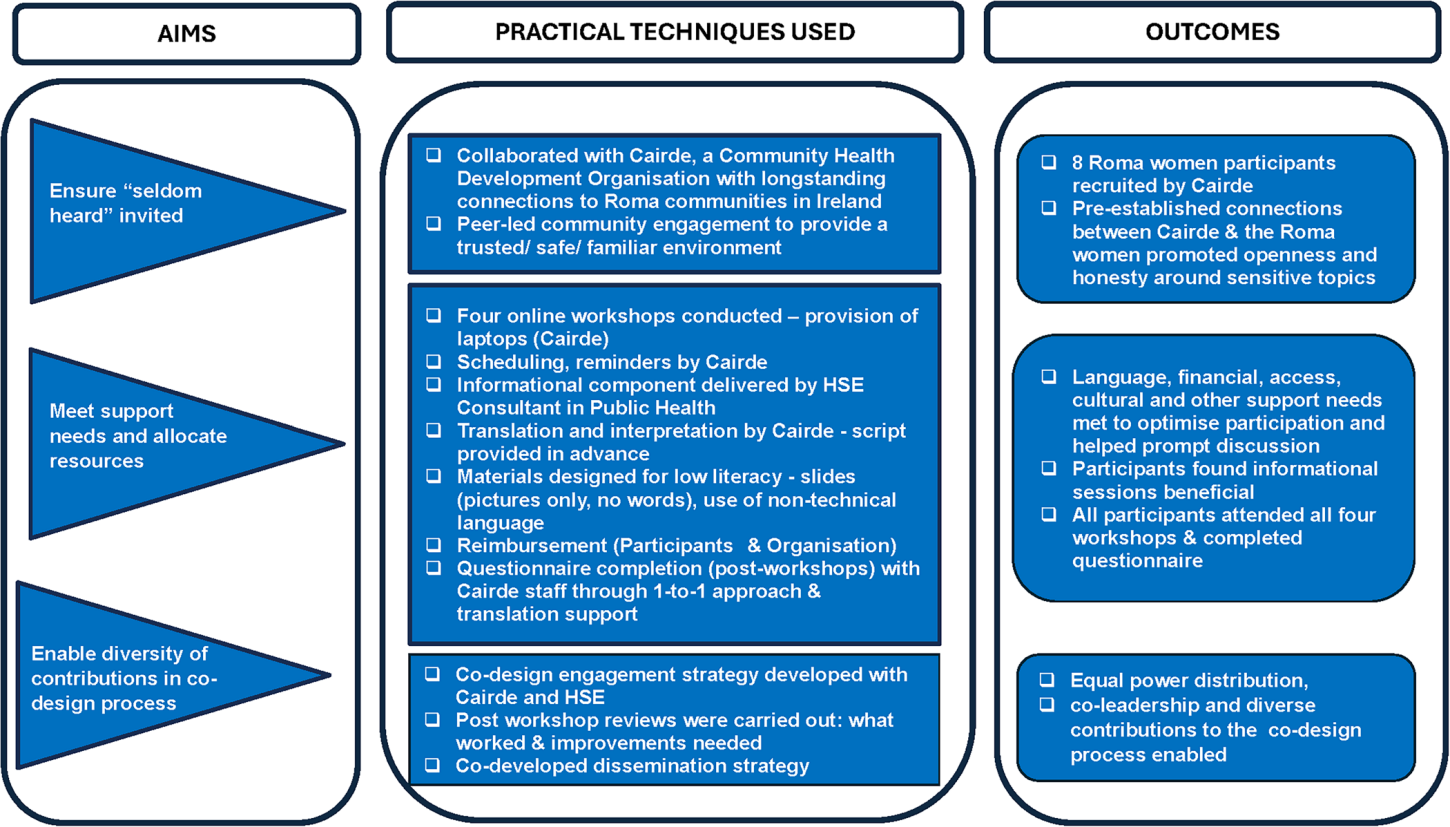
Optimising co‐design with ethnic minority communities (Chauhan et al. 2021).

The co‐design process (Figure 1) aligns with three key areas identified by Chauhan et al. (2021). To optimise the engagement process with the Roma community and its potential to lead to the desired outcomes, this study firstly aimed to invite seldom‐heard women who have engaged with maternity services in Ireland to participate in the co‐design work; secondly, appropriate resources were provided to support participation through the co‐design process; and finally, diverse contributions were supported and enabled as part of the co‐design process [31].

### 2.1 Ensure ‘Seldom‐Heard’ Invited and Included in Co‐design Work

At the beginning of this study, the Research Team approached Cairde, a community health development organisation, to discuss their interest and capacity to be involved in this study. While Cairde were known to the research team, this study was the first time the research team worked with the organisation. Cairde run a series of supports and projects with the aim of reducing health inequities among minority ethnic communities [32]. Through Cairde's work with Roma communities spanning over 15 years, the organisation has built strong links with the community. Cairde's Roma Programme is dedicated to promoting the health, equity, and inclusion of Roma communities in Ireland. Through community development, advocacy, and culturally responsive support, the programme works to reduce health inequities, combat discrimination, and empower Roma individuals to access their rights and participate fully in Irish society [33].

Following their agreement to participate, a series of meetings were held between Cairde, the research team and staff from the HSE to discuss how the engagement might be co‐designed to benefit members of the Roma community and effectively engage with Roma women to capture their experience of maternity services in Ireland. The workshop structure, content and methodology were devised at these meetings. The Cairde team included two Roma peer support workers who advised on cultural considerations and provided insight on the extent of literacy challenges. They participated in discussions on the broad topics to be discussed in the FGDs, but did not have a role in the design of individual questions. Both women were participants in the workshops and FGDs, also acting as translators. Roma women who had given birth in an Irish hospital in the previous 5 years were deemed eligible to participate. Recruitment was led by Cairde from their service users, and eight women were recruited to participate in the project.

The aim of the FGDs was to ensure that reported measures of quality and safety reflect the experience of the Roma women using maternity services. Achieving this aim required balancing the gathering of data on the experiences and perspectives of participants in a structured manner with a workshop structure that allowed participants with literacy and language limitations to speak freely and contribute to the process. An online workshop format was agreed and a series of three initial workshops and one online questionnaire with two parallel objectives were co‐designed with Cairde staff. The objectives were as follows:

i. to capture the experiences and perspectives of Roma women of pregnancy, childbirth, and postnatal care in Ireland
ii. to provide information on pregnancy, childbirth, and postnatal care services and how to care for themselves and their baby.

The online format was considered preferable as in‐person attendance was challenging for participants, as most had young children. Cairde ensured all participants had access to and proficiency in the necessary technology. The information component of the workshops was considered important by Cairde to help address the known gaps in knowledge and to promote engagement by Roma women with maternity services, in particular during the antenatal period. The information sessions were delivered by a Health Service Executive (HSE) Public Health doctor and the inclusion of this component ensured reciprocity in the partnership. Participants were provided with information on pregnancy and childbirth, the programme of care while pregnant (Workshop 1), maternity care available during labour, birth and while in hospital (Workshop 2), and postnatal care available for themselves and their baby (Workshop 3), allowing the participants to discuss how their care experience compared to the information provided.

### 2.2 Meet Support Needs and Allocate Resources

The co‐designing process with Cairde and members of the Roma community identified a number of potential barriers to engagement, and the engagement process was designed to address these barriers.

Participants had varying levels of proficiency in English in particular, and literacy in general, some with none and others with varying levels of spoken and written English. Following a search of the literature, several interaction methods were reviewed to facilitate engagement in the workshops. Particular consideration was given to group work methods suggested by Liberating Structures such as *User Experience Fishbowl* and *Heard, Seen, Respected* [34]. However, it was decided that the need to incorporate translation and interpretation added complexity to these strategies, potentially distracting from achieving the objectives of the engagement. As FGDs are useful in health services research with minority groups ‘whose voices have been otherwise muted’ [35, 36], this methodology was adopted. Breakout rooms were used to reduce the size of the overall group and to encourage all women to contribute their own experiences. As the proposed suite of quality metrics included measures related to antenatal, maternal and neonatal care, infant feeding, and health service management, workshops were structured across these topics and questions were drafted to discuss participants' experience of care. The number of predefined questions was kept to a minimum to allow free flow of discussion and to allow the facilitator to probe where necessary (Supporting Information: Appendix B).

Each online workshop was planned for 2 h in duration, commencing with an informational component followed by FGDs on topics relevant to the informational component delivered. FGDs were led by two researchers experienced in delivering online workshops, who asked the women to share their experiences, encouraged discussion, and individually invited all participants to contribute with the support of the Roma peer support workers, who also provided translation and interpretation in Romani when needed. The information component delivered by a Public Health doctor used slides depicting topics in pictures and graphics only (Supporting Information: Appendix A), without words, and the script for each session was provided in advance to Cairde staff. The information component also invited questions from participants. A post‐workshop debrief was scheduled with Cairde staff, including the two Roma peer support workers, directly after each workshop to agree refinements in the approach where necessary.

Workshops were scheduled weekly over a 3‐week period online in the morning while the Roma women's older children were at school. The weekly scheduling was agreed to ensure continuity in the process. It was also agreed that FGDs would be audio recorded for transcription purposes and recordings subsequently destroyed to protect the anonymity of participants. Minutes and audio recordings of all workshops were shared with Cairde for review purposes.

Three days before the commencement of the first workshop, in compliance with the ethical approval granted by the university and the HSE, all participants were asked to read a participant information leaflet (PIL) (Supporting Information: Appendix D) and sign a consent form (Supporting Information: Appendix E), both co‐designed with Cairde to ensure the language was accessible to those with low levels of literacy and sensitive to Roma culture. All participants were supported by Cairde staff in reviewing the PIL and consent form to ensure they understood the purpose of the engagement, the process and the time commitment. Translation and interpretation support was provided by the Roma peer support workers, who were well known to participants. It was agreed with Cairde that participants would be asked if they understood the consent form and invited to ask questions at the beginning of the first workshop.

Once all workshops were complete, all participants were asked to answer a co‐designed questionnaire (Supporting Information: Appendix C). This online questionnaire captured data about the participants' backgrounds and their utilisation of healthcare during pregnancy, childbirth and postnatal period. The questionnaire also allowed participants to provide further information about their experience of the quality of maternity services that they may not have expressed in the FGDs context.

The workshops were delivered using infrastructure, such as laptops, Zoom licenses, and other resources, provided by Cairde's Roma Programme. The payment made by the research team for this project was specifically intended to engage a targeted group of women among Cairde's Roma service users and to compensate them for the time they spent participating in the workshops and completing the questionnaires. The Roma peer support workers who participated in the co‐design were paid by Cairde and the co‐design formed part of this role.

### 2.3 Support and Enable a Diversity of Contributions via the Co‐design Process

A key objective was to develop a mutually beneficial research partnership between the academic researchers, collaborators within the HSE and Cairde, capable of enduring beyond this study. A sustained relationship requires trust and equal power distribution.

Roma peer support workers working with Cairde engaged in the planning and delivery of the workshops. They acted as community leaders, recruiting participants, promoting attendance and providing translation and interpretation support. After each workshop, a debrief session was held between the academic researchers, staff from the HSE, and Cairde, including the Roma peer support workers, to assess how the workshop went and to discuss refinements to the approach for the next workshop. A dissemination strategy was jointly agreed to reflect the objectives of all parties to the research. The strategy included feedback and discussion of findings with Cairde and joint authorship.

## 3 Results

All participants attended all online workshops (*n* = 8), and Table 1 outlines the characteristics of participants.

**Table 1:** Characteristics of workshop participants.

	No. of participants
**Age (in years)**	
20–25	1 (12.5%)
26–30	2 (25%)
31–35	3 (37.5%)
Above 35	2 (25%)
**Number of years lived in Ireland**	
Less than 5 years	3 (37.5%)
5–15 years	4 (50%)
More than 15 years	1 (12.5%)
**No of children**	
1–2	1 (12.5%)
3–4	2 (25%)
5 and more	5 (62.5%)
**Age at the time of first pregnancy**	
Less than 15 years	1 (12.5%)
15–18 years	6 (75%)
More than 18 years	1 (12.5%)
**Place of Birth**	
Romania	7 (87.5%)
Other	1 (12.5%)
**Level of English (understanding and speaking)**	
Very Well	2 (25%)
Well	3 (37.5%)
Not well or not at all	3 (37.5%)
**Level of English (reading)**	
Very Well	Nil
Well	3 (37.5%)
Not well or not at all	5 (62.5%)

Most participants had large families and gave birth to their first child before the age of 19. English proficiency was limited, with many requiring support.

Cairde handled all administrative tasks with the participants, including scheduling, payments, and supporting those with literacy and language needs in the completion of paperwork. Online delivery was critical to both recruitment and maintaining engagement and was made possible by the provision of laptops and mobile phones to participants by Cairde. Where needed, Cairde staff contacted participants to assist with connecting to the online workshops, which supported the continued engagement of all participants. The remuneration of workshop attendees was also likely to have encouraged attendance.

The involvement of Cairde's Roma peer support workers helped ensure the cultural appropriateness of the discussions and put participants at ease. The level of engagement in the FGDs was high, and the women spoke openly about their experiences and advocated for improvements required in healthcare service delivery. For example, participants expressed the lack of culturally appropriate interpreter support in maternity services, i.e., a female interpreter from the Roma community, significantly impacted their experience of the quality of healthcare received. Workshop participants were already known to each other, encouraged each other to speak and drew on each other's experiences. Reflecting on her experience of co‐designing and participating in this study, one of the Roma peer support workers explains the importance of including community members, ‘one of them’, in research design and delivery:

*Before the workshops, I didn't fully understand how the maternity system worked in Ireland, even though I had already gone through it myself. Being both a participant and an interpreter gave me a unique role. I wasn't just translating words; I was helping to give voice to real experiences that often go unheard. While supporting other Roma women to understand the information, I was also learning about our rights and the kind of care we should expect. Because I am also Roma, and a mother, the women saw me as one of them and it made it easier for them to trust me and to speak openly. That kind of connection and trust is only possible when the interpreter understands the community from the inside. The use of pictures and our own language made a huge difference. Because the group was safe and familiar, the women felt comfortable sharing things they rarely talk about. It felt like our stories were truly valued. This project showed me that our voices matter and that we can help change the system, not just for Roma women, but for everyone.*

With the benefit of hindsight, the slides for the first online workshop did include some limited text, and some technical terms such as ‘metrics’ or ‘indicators’ as the extent of the literacy challenges were not fully appreciated. Learnings from this first workshop, following the postworkshop debrief, resulted in a refinement of the approach for all remaining workshops, with the presentation of slides using pictures and graphic only, and the careful adoption of nontechnical language.

**Figure 2:**
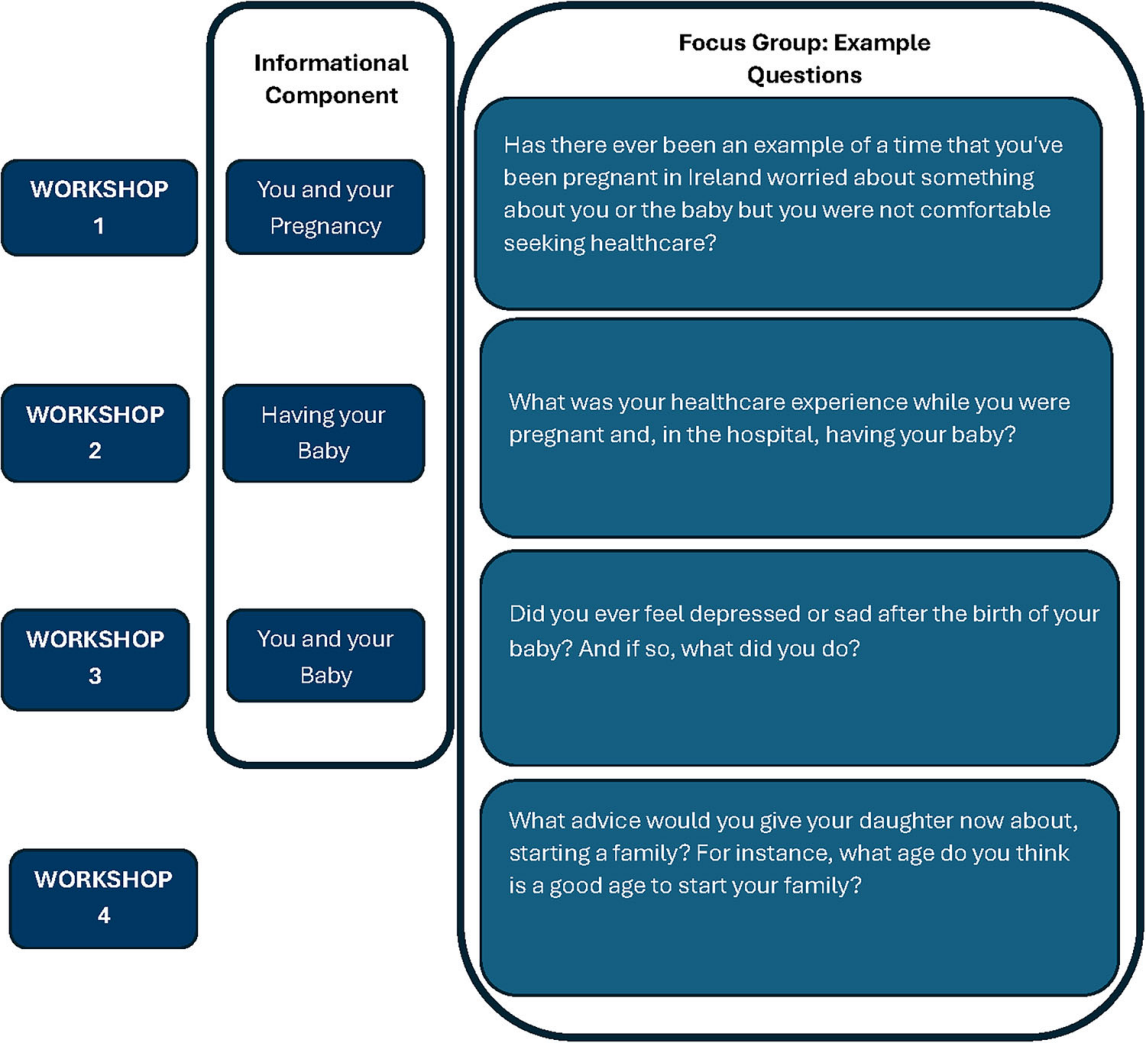
Workshops: informational component and example questions.

The 2‐h workshops were initially designed to allow 40 min for the maternal health information component, 20 min each for 2 FGDs to be held in breakout rooms, and 20 min for feedback discussions following the breakout rooms. However, during the first two workshops, the delivery of the maternal health information required over an hour due to the time taken to facilitate translation and interpretation. Therefore, during the debrief following the second workshop, it was agreed that an additional fourth workshop was required without an informational component to ensure all participants had sufficient time to discuss their experiences relevant to all topics (Figure 2). Workshops were held over a 4‐week period running from late‐May to mid‐June 2024, including this additional fourth workshop.

All participants completed the questionnaire with the assistance of Cairde's staff, including a Roma peer support worker when translation and interpretation support was needed.

Cairde's staff, who are familiar with the women and their family make‐up, were able to offer clarification specific to the women's circumstances where necessary. While this supported approach to data collection is not usual, it was of great value in ensuring full and accurate responses.

The primary aim of the research was to inform the development of measures of quality of maternity and neonatal services, and findings allowed the relevance of a proposed suite of measures to be critically assessed to ensure they adequately captured the experiences identified through this engagement. The Roma women participating in this study found their encounters with the health services to be complex and challenging. Participants faced challenges in accessing maternity and social services due to low health literacy, previous experience and their cultural preferences. They reported difficulty at every level, from engaging with healthcare services to applying for social support and child benefits. While awareness of free maternity care and antenatal services was low during their first pregnancies, postnatal care was more consistently accessed. The detailed findings are presented in a companion paper [37].

It was also deemed important to ensure the research and engagement contributed to supporting service improvements for Roma women, and opportunities for continued engagement were identified.

## 4 Discussion

A collaborative rapid realist review conducted by Ní Shé et al (2019) found that undertaking reciprocal PPI with seldom‐heard groups often requires heroic efforts from all parties involved, and sometimes places undue burden on community partners to enable research at the eleventh hour and ensure the project is culturally appropriate and accessible [28]. However, this study clearly demonstrates that establishing a strong partnership with a community organisation that has proven expertise and trusted relationships is essential — and that careful preparation and planning can only be effective when built on this foundation. There is a need for targeted approaches to involve specific groups, including marginalised groups, to overcome barriers of low literacy, communication and language difficulties. The co‐design approach adopted in this engagement ensured the online workshops were designed to address barriers to accessibility, relying on pictures and verbal communication, the use of non‐technical language, and translation and interpretation by Cairde's Roma peer support workers.

As reported by Ocloo et al. (2021), barriers to engagement include accessibility and scheduling of meetings [38, 39], limited time to be involved [38, 39, 40, 41, 42], dealing with the paperwork [39] and transportation [26]. The existing provision of laptops, phones and training in using this technology by Cairde, meant a cohort of women from a marginalised community could participate without the need to arrange transport and childcare.

Collaborative approaches can address paternalism and power relations and help with self‐reflection and co‐publication [43]. Understanding the difference in perspectives and needs of community organisations, building trust and creating capacity building opportunities are important steps for researchers to consider towards building durable partnerships [44]. One of the most novel components of this study is the reciprocal nature of knowledge sharing, with participants, researchers, Cairde and healthcare providers all contributing their knowledge and learning from each other, promoting equal distribution of power and building trust. Designing the information component within the workshops allowed Cairde to incorporate this study within their existing programme, and the HSE team to provide information on available health and wellbeing services to a marginalised population who are often hard to reach. The information component also allowed the FGDs to be framed against existing service provision.

Partnership with Cairde staff (M.P., E.M., C.D., D.D.) was pivotal to understanding and gaining the trust of the Roma women participating in this study. None of the research team are from an ethnic minority background and none are economically disadvantaged. Two of the three academic researchers are mothers and accessed maternity care in Ireland (T.Mc.D., E.Mc.A.). One of the researchers is a midwife with experience of delivering maternity care in India (J.K.D.). None of HSE staff who engaged in the co‐design and delivery of this study are responsible for the delivery of maternity or neonatal care. L.H. and G.M. are also mothers with their own experience of maternity care in Ireland.

The need to compensate participants is often raised when engaging patients and the public in research [22, 26]. Remunerating those engaging with this study ensured Cairde could recruit and maintain engagement, and reimbursement of tutor support costs to Cairde ensured they could provide the level of support needed to complete the questionnaire. Making funding resources available to community partners to facilitate engagement within community spaces was an important mechanism identified by the Ní Shé et al (2019) review to enable seldom‐heard participation [28].

While the endurance of the partnership will be determined in the future, this engagement process and the joint dissemination strategy has increased trust in the HSE and laid the groundwork to extend the collaboration for the benefit of the community.

## 5 Strengths and Limitations

The combined use of workshops for both health information and the research process was novel. The reciprocity of the relationship held the participants' interest and gained their trust. Structuring the workshops according to the maternity and birth journey provided a suitable chronological format to the informational aspects and this helped guide the FGDs.

Working with Cairde was a major strength in this study process: they had pre‐established access and knowledge of the Roma women. It is unlikely the women would have engaged with the research team had they been approached directly. The staff at Cairde have an in‐depth understanding of the nature of the Roma women's marginalisation and life experiences. They were able to convey this during the research process, without divulging any individual personal circumstances, which was invaluable to the research team. Furthermore, payment also ensured participation and supported the engagement of Cairde's staff. It is unlikely that all women would have managed to answer the questionnaire without this support, as literacy and language challenges would have been an insurmountable barrier for some participants if it had been self‐administered. Assistance in completing the questionnaire is also likely to have improved validity and response rate. Using an online approach enabled the research team to reach the Roma community in a way that an in‐person research approach would not have. Provision of laptops/mobile phones was essential and ensured participation.

There were some limitations to the approach adopted in this study. Language differences were a challenge to the flow of the workshops at times. Also, as members of the research and health service teams did not speak Romani, significant reliance was placed on interpretation by the Roma peer support workers. Furthermore, participants did express their preference for the online format over in‐person engagement during the FGDs. However, this may not have been the case for all women. While the online approach did ensure participation, reducing barriers like the need for childcare and travel, online engagement may miss the personal interactions and non‐verbal cues that flow from in‐person engagement. Many aspects of this approach may be generalisable to engaging other minority communities; however future research should involve a larger, more diverse sample to strengthen findings. Finally, methods adopted are heavily reliant on a representative organisation that is motivated to invest time and resources in building relationships and trust. This process also required significant time investment by all parties, from the initial engagement, the co‐design process, workshop and questionnaire delivery, and interpretation of findings, which may be challenging for some organisations. Many community organisations lack the necessary funding to participate meaningfully in research activities. It is therefore essential that research projects include dedicated funding for community organisations when their involvement is central to the study.

## 6 Conclusion

Through partnering with a community health development organisation, a research collaboration with academic researchers and the health service captured the experience and perspectives of the quality of maternity services by Roma women [37]. The many barriers to this engagement were addressed by identifying and partnering with Cairde – an organisation with strong links to the Roma community and with the capacity to participate. Partnering with Cairde afforded privileged access to the Roma community, allowing the research team to engage with this seldom‐heard group. Future research should focus on expanding representation and designing and evaluating community‐based health initiatives.

## Author Contributions

**Thérèse McDonnell:** conceptualisation, project administration, methodology, investigation, formal analysis, visualisation, writing – original draft preparation, writing – review and editing. **Jaspreet Kaur Dullat:** conceptualisation, methodology, investigation, formal analysis, visualisation, validation, writing – original draft preparation, writing – review and editing. **Louise Hendrick:** resources, validation, methodology, writing – review and editing. **Léan McMahon:** writing – review and editing. **Gemma Moore:** validation, writing – review and editing. **Marianna Prontera:** project administration, resources, methodology, validation, writing – review and editing. **Emily Murphy:** resources, project administration, methodology, validation, writing – review and editing. **Carmen Nae:** resources, project administration, validation. **Danut Nae:** resources, project administration. **Eilish McAuliffe:** conceptualisation, formal analysis, funding acquisition, writing – review and editing.

## Ethics Statement

This study has received ethical approval from UCD Human Research Ethics Committee‐ Sciences (Reference Number: LS‐LR‐23‐184‐McAuliffe) and the Research Ethics Committee Midlands Area and Corporate (Regional Health Area B) of the Health Service Executive (Study: Evaluation of development and proposed implementation of the Quality and Safety Signals Proof of Concept in Maternity and Neonatal Services, Ref: RRECB1123LH).

## Conflicts of Interest

The authors declare no conflicts of interest.

## Supporting information


**APPENDIX A:** EXAMPLE WORKSHOP SLIDE & SCRIPT.
**APPENDIX B:** FOCUS GROUP QUESTIONS.
**APPENDIX C:** QUESTIONNAIRE.
**APPENDIX D:** PARTICIPANT INFORMATION LEAFLET.
**APPENDIX E:** CONSENT FORM.

## Data Availability

The anonymised transcription of the focus groups that support the findings of this study are available on reasonable request from the corresponding author.
